# YTHDF2 Suppresses Notch Signaling through Post-transcriptional Regulation on Notch1

**DOI:** 10.7150/ijbs.61573

**Published:** 2021-08-28

**Authors:** Byongsun Lee, Seungjae Lee, Jaekyung Shim

**Affiliations:** Department of Bioresources Engineering, Sejong University, Seoul 05006, Republic of Korea

**Keywords:** YTHDF2, Notch signaling, m^6^A

## Abstract

YTH domain family 2 (YTHDF2) is an N6-methyladenosine (m^6^A) binding protein promoting mRNA degradation in various biological processes. Despite its essential roles, the role of YTHDF2 in determining cell fates has not been fully elucidated. Notch signaling plays a vital role in determining cell fates, such as proliferation, differentiation, and apoptosis. We investigated the effect of YTHDF2 on Notch signaling. Our results show that YTHDF2 inhibits Notch signaling by downregulating the *Notch1*, *HES1*, and *HES5* mRNA levels. Analyzing YTHDF2 deletion mutants indicates that the YTH domain is critical in regulating the Notch signal by directly binding m^6^A of *Notch1* mRNA. Recently, YTHDF2 nuclear translocation was reported under heat shock conditions, but its physiological function is unknown. In our study, the YTH domain is required for YTHDF2 nuclear translocation. In addition, under heat shock stress, the Notch signal was significantly restored due to the increased expression of the Notch1 targets. These results suggest that YTHDF2 in the cytoplasm may act as an intrinsic suppressor in Notch signaling by promoting *Notch1* mRNA degradation under normal cellular conditions. Conversely, upon the extracellular stress such as heat shock, YTHDF2 nuclear translocation resulting in reduced *Notch1* mRNA decay may contribute to the increasing of Notch intracellular domain (NICD) regulating the survival-related target genes.

## Introduction

The YT521-B homology (YTH) domain was identified by sequence comparison and found in 174 different proteins expressed in eukaryotes 1. The YTH domain-containing proteins are actively involved in transcripts such as mRNA splicing, mRNA stability, translation process, and epigenetic gene expression control 2, 3. The YTH domain serves as the module for recognizing N^6^-methyladenosine (m^6^A). There are five YTH domain-containing proteins in humans, namely, YTHDC1, YTHDC2, YTHDF1, YTHDF2, and YTHDF3 4. YTHDF2, which induces destabilization of m^6^A containing RNA, is actively studied as a significant m^6^A reader protein 5. Most research on YTHDF2 focused on the regulation of RNA stability and processing. m^6^A of RNA in the cells regulates mRNA stability, mRNA translation, microRNA regulation, and pre-mRNA 5, 6. m^6^A formation is catalyzed by m^6^A writer^7^, ^8^, several methyltransferases complex including METTL3, METTL14, and WTAP 8. The number of m^6^A in RNA is also determined by the demethylase function of m^6^A erasers such as FTO and ALKBH5 9, 10. m^6^A s of various consensus in RNA is selectively recognized by major m^6^A reader proteins such as YTH domain-containing proteins, hnRNPs, and eIF3 11. Different m^6^A reader proteins containing the YTH domain bring up the final RNA destabilization factors to determine the appropriate transcript dosage in the cell 12. YTHDF2- m^6^A RNA complexes exist in various forms in all intracellular RNA types and function as mRNA stability regulators in the cytoplasm 5, 13.

Despite its essential role, studies on the effects of YTHDF2 on regulating several key signaling pathways that determine cell fate are insufficient.

Notch signaling, one of the evolutionary well-conserved signaling pathways, is critical for embryo development and the progress of various cancers. 14. The Notch is composed of its substrate, Jagged or Delta, and its receptor, the Notch 1-4 family, and the primary characteristic mechanism is the signaling between cells 15. One cell acts as a signal sending cell that expresses an excess of the substrate on the cell membrane, while the other cell acts as a signal receiving cell that expresses a receptor on the cell membrane 15. Notch bound to the ligand in the cell membrane is cleaved by γ-secretase and convertase 16. The released NICD moves to the nucleus, regulating gene expression by activating the transcription factor CSL. In this regard, in our previous work, the movement restriction of NICD to the nucleus by the nuclear membrane protein emerin has been identified as a potential Notch signaling regulation 17. Nuclear NICD binds with various proteins such as RBP-J and mastermind to promote specific gene transcription 18. These genes encoding basic HLH are transcriptional repressors that influence cell proliferation and differentiation. The mammalian hairy enhancer of split 1 (Hes1) and Hes5 inhibits cell differentiation in embryogenesis 19. The Notch signal is characterized by a self-amplified mode targeting the *Notch1* gene 20. This mode is intended to rapidly respond to various external stimuli and eventually activate cell proliferation signals.

As a related study on YTHDF2 and Notch signaling, the report states that YTHDF2-mediated Notch1a mRNA destruction during zebrafish embryo development contributes to regulating hematopoietic stem cells and progenitor cells specification 21. We analyzed the homology of *Notch1* mRNA and YTHDF2 protein in humans and Zebrafish using Clustal omega analysis. As a result, *Notch1* mRNA showed 72% homology (Supplementary Table 1), and YTHDF2 showed 70.7% homology (Supplementary Table 2).

Based on these clues, we investigated the possibility of YTHDF2 regulation on Notch signaling caused by various external stimuli in human cells. This study focused on the YTHDF2 function as an intrinsic Notch signal suppressor promoting *Notch* mRNA degradation. We found the YTH domain of YTHDF2 is critical for the suppression of Notch signaling by destabilizing *Notch1* transcripts. Recently, heat shock stress-induced YTHDF2 migration to the nucleus was reported 22. We investigated the effect of YTHDF2 nuclear translocation on the Notch signal in such a condition. The Notch signal was significantly restored due to increased Notch target gene expression under heat shock stress. These results suggest that Notch signaling plays an essential role in cell survival and proliferation by avoiding the inhibitory action of YTHDF2 even under extracellular stress conditions such as heat shock.

## Materials and Methods

### Cell culture and transfection

HeLa cells (American Type Culture Collection, Manassas, VA, USA) were cultured in DMEM (HyClone, Logan, UT, USA) supplemented with 10% FBS (HyClone, Logan, UT, USA) and Antibiotic antimycotic solution (Corning, Manassas, VA, USA) at 37°C with 5% CO_2_. Jurkat cells (American Type Culture Collection, Manassas, VA, USA) were cultured in RPMI (HyClone, Logan, UT, USA) supplemented with 10% FBS (HyClone, Logan, UT, USA) and Antibiotic antimycotic solution (Corning, Manassas, VA, USA) at 37°C with 5% CO_2_. Transfection was performed using the Lipofectamine 2000 reagent (Invitrogen, Grand Island, NY, USA) according to the manufacturer's instruction. The transfected cells were cultured for 24-48 h, washed with DPBS, and harvested with lysis buffer (#FNN0011; Life technology, Grand Island, NY, USA).

### Antibodies

For immunoblotting, primary antibodies specific for YTHDF2 (1:5000, #RN123PW), β-actin (1:5000, #M177-3), Myc (1:5000, #M192-3) were purchased from MBL (MBL, Woburn, MA, USA). NICD antibody (1:3000, #07-1231), HES1 (1:1000, #ab 5702) and HES5 (1:1000, #ab 5708) were obtained from EMD Millipore Corporation (EMD Millipore Corporation, Temecula, CA, USA). Primary antibodies specific for Flag (1:5000, #F7425) were purchased from Sigma-Aldrich (Sigma-Aldrich, St. Louis, MO, USA). GAPDH antibody (1:5000, #AM4300) was obtained from Applied Biological Materials (Applied Biological Materials, Richmond, BC, Canada). FITC (1:500, # 209-095-082) and TRITC (1:500, # 209-025-082) antibodies were purchased from Jackson Immuno Research Laboratories (Jackson Immuno Research Laboratories, West Grove, PA, USA). m^6^A antibody (#A-1801) was purchased from EpiGentek (EpiGentek, Farmingdale, NY, USA).

### Plasmid constructs

Human YTHDF2 and NICD cDNA were provided by 21C Frontier Human Gene Bank (Seoul, South Korea). The YTHDF2 cDNA was amplified by PCR and inserted into the restriction enzyme sites of pEGEP-C1 for biochemical studies. The amplified full-length NICD cDNA was inserted into the restriction enzyme sites of pEGFP-C1 (Clontech, Mountain View, CA, USA) for immunocytochemistry. Alternatively, both amplified genes were inserted into the restriction enzyme sites of pcDNA3 for biological assays. For the construction of YTHDF2 deletion mutants or NICD deletion mutants, the corresponding regions were amplified by PCR and inserted into the restriction enzyme sites of HA-pcDNA3. YTHDF2-Flag was provided by Addgene (Addgene, Watertown, MA, USA), and another NICD constructs were kindly provided by Dr. HS Park (Chonnam National University, South Korea).

### Immunocytochemistry

HeLa cells were plated on glass coverslips and then transfected with 0.5 μg of vectors or heat stress (42°C, 1h). After incubation, cells were fixed with 4% paraformaldehyde, permeabilized with 0.1% Triton X-100 in PBS, and then incubated with a blocking solution (Dako, Glostrup, Denmark). After incubation overnight with primary antibody for NICD (1:200, Millipore, Billerica, MA, USA) or YTHDF2 (1:200, MBL, Woburn, MA, USA) in blocking solution, cells were washed and incubated with FITC or TRITC-conjugated secondary antibodies (1:200, Jackson Immuno Research Laboratories, West Grove, PA, USA) for 1 h at room temperature. After staining with DAPI (Life Technologies, Carlsbad, CA, USA), cells were observed under a confocal microscope (Leica TCS SPE, Buffalo Grove, IL, USA).

### m^6^A RNA Methylation Quantification Kit

Total RNA was isolated using Trizol reagent (Life Technologies, Life Technologies, Carlsbad, CA, USA), and 0.2 μg of total RNA was used for m^6^A RNA Methylation Quantification Kit. We were performed according to the manufacturer's protocol (#P-9005-96; Epigenetics, Farmingdale, NY, USA).

### Cellular fractionation and immunoblotting

Cellular fractionation was performed as described previously 23, 24. Briefly, cells were washed twice with cold PBS, harvested by scraping, and incubated in hypotonic buffer (20 mM HEPES, pH 7.0, 10 mM KCl, 2 mM MgCl_2_, 0.5% NP-40, 1 mM Na_3_VO_4_, 10 mM NaF, 1 mM phenylmethanesulfonyl fluoride, 2 μg/ml aprotinin). After 10 min of incubation on ice, cells were homogenized by 15-20 strokes in a Dounce homogenizer. The homogenate was centrifuged for 5 min at 1,500×*g* to sediment nuclei. The supernatant was then centrifuged at 16,000×*g* for 20 min to separate cytosolic and total membrane fractions. After washing the nuclei pellets three times with hypotonic buffer, the pellets were incubated with lysis buffer and centrifuged at 16,000×*g* for 20 min to extract nuclear proteins. To obtain total lysates, cells were incubated with the lysis buffer and centrifuged at 16,000×*g* for 20 min. SDS-PAGE resolved proteins and transferred them onto PVDF membranes (Millipore Corporation, Temecula, CA, USA). Membranes were blocked for 1 h at room temperature with a 5% nonfat milk powder solution or 3% BSA in TBS containing 0.05% Tween-20 (TBST). The membranes were then incubated with primary antibody in blocking solution overnight at 4°C. The membranes were washed three times with TBST and incubated with secondary antibody for 1 h at room temperature. After washing three times with TBST, the membranes were developed using the ECL detection system (Bio-Rad, Hercules, CA, USA).

### Quantitative RT-PCR (qRT-PCR)

Total RNA was isolated using the Trizol reagent, and 1 µg of total RNA was used for cDNA synthesis. cDNA was amplified using primer pairs for human *Notch1* (forward 5′-TACGTGTGCACCTGCCGGG-3′, reverse 5′-CGTTTCTGCAGGGGCTGGGG-3′), human *HES1* (forward 5′-ATGACGGCTGCGCTGAGCAC-3', reverse 5'-TAACGCCCTCGCACGTGAC-3'), human *HES5* (forward 5′-CCGGTGGTGGAGAAGATG-3′, reverse 5′-GACAGCCATCTCCAGGATGT-3′), and human *GAPDH* (forward 5′-GTCGGAGTCAACGGATTTGG-3′, reverse 5′-AAAAGCAGCCCTGGTGACC-3′). *YTHDF2* primers (#P320237V) and *METTL3* primers (#P204985V) were obtained from Bioneer (Bioneer, Daejeon, South Korea). qRT-PCR was performed using the StepOne Real-Time PCR System (Applied Biosystems, Foster City, CA, USA). Reactions were amplified using the selective primers described above and an EvaGreen 2× qRT-PCR MasterMix-iCycler (ABM) according to the manufacturer's instruction.

### Small interfering RNA (siRNA)

YTHDF2 (#51441), METTL3 (#56339), or negative control (#SN-1002) siRNA oligonucleotides were purchased from Bioneer (South Korea). Transfection was performed with Lipofectamine RNAiMAX reagent (Life Technologies) in HeLa cells according to the manufacturer's protocol. The nucleotide sequences for siRNA targeting were as follows: YTHDF2 (sense sequence, 5′-GUGCAUACAGUUUUCUA-3′ and antisense sequence, 5′-UAGAGAAAACUGUAUGCAC-3′), METTL3 (sense sequence, 5'-GUGCAACCCAACUGGAUCA-3' and antisense sequence, 5'-UGAUCCAGUUGGUUGCAC-3'). Negative siRNA was non-targeting siRNA for human, mouse, and rat.

### RNA immunoprecipitation (RIP)

RIP experiments were performed using containing RNA silencing HeLa cells. Cells were treated formaldehyde drop-wise directly to the media to a final concentration of 0.75% and rotate gently at room temperature (RT) for 10 min. Add glycine to a final concentration of 125 mM to the media and incubate with shaking for 5 min at RT. Cells were rinsed twice with 10 ml cold PBS, added 5 ml of cold PBS, scraped thoroughly with a cell scraper, and transferred into 50 ml tube. Collected cells were centrifuge for 5 min, 4°C, 1,000 x g and aspirate off supernatant and suspend the pellet in freshly prepared 1mL RIP buffer (150 mM KCl, 25 mM Tris (pH 7.4), 5 mM EDTA, 0.5 mM DTT, 0.5% NP40, 100 U/mL RNAase inhibitor, Protease inhibitors). The suspended pellet was shear chromatin using a Dounce homogenizer with 15-20 strokes. The homogenate was centrifuged at 13,000 rpm for 10 min. The supernatant was added antibody and incubated overnight at 4°C with gentle rotation. Supernatant added protein A/G beads (40 µl) and incubated for 2 h at 4°C with gentle rotation. Pellet beads at 2,500 rpm for 30 s, remove supernatant and resuspend beads in 500 μl RIP buffer. Resuspended beads were washed repeat for a total of three RIP, followed by one wash in PBS. Isolate coprecipitated RNAs by resuspending beads in 1ml TRIzol RNA extraction reagent (1 ml) according to manufacturer's instructions. 1µg of RNA was reverse-transcribed using the SuperScript IV kit (Invitrogen) followed by qPCR using EvaGreen 2× qRT-PCR MasterMix-iCycler (Applied Biological Materials, Richmond, BC, Canada) according to the manufacturer's instruction.

### Analysis of mRNA degradation

HeLa cells were treated with 10 µg/ml of actinomycin D (Enzo Life Sciences) in the YTHDF2 or YTHDF2 mutants. Total RNA was extracted using TRIzol reagent according to the manufacturer's instructions. qRT-PCR was then performed as previously described.

### Statistical analysis

The results are presented as the mean ± S.D. Statistical significance was determined with the Student's *t*-test with a significance level of P <0.05. The data for the transcription PCR array were presented as the mean of two independent experiments.

## Results and Discussion

### YTHDF2 inhibits the expression of genes downstream of Notch signaling

It is well known that YTHDF2 recognizes and regulates m^6^A in RNA. However, the exact function related to intracellular signaling has not been fully elucidated. For that reason, we investigated the possibility of YTHDF2 regulation on the Notch signaling pathway, one of the critical signaling determining cell fates.

**Figure 1:**
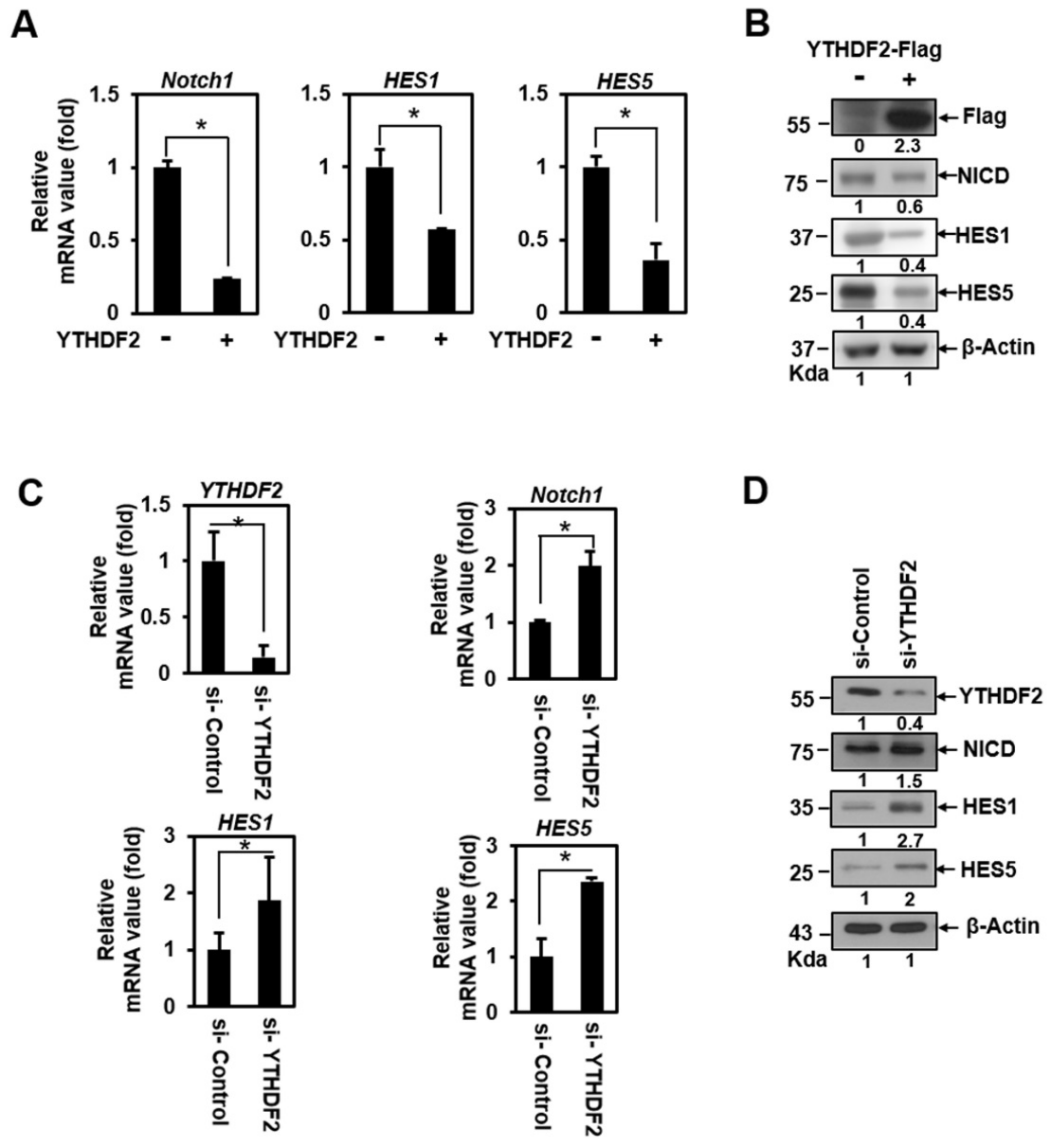
YTHDF2 downregulates the Notch1 signal. A & B. HeLa cells were transiently transfected with vectors encoding the YTHDF2. Total RNA was isolated and subjected to qRT-PCR analysis, and transfected cells were lysed and subjected to Western blotting with antibodies against Flag, NICD, HES1, HES5, and β-actin. The membranes were analyzed using ImageJ software (NIH, Bethesda, NY, USA), and qRT-PCR data were normalized to *GAPDH* expression. The results represent the mean ± S.D. of three independent experiments performed in triplicate. *, P <0.05. C. HeLa cells were treated with siRNA (100nM) against YTHDF2 (si-YTHDF2) or control (si-Control) for 48 h. Total RNA was isolated and subjected to qRT-PCR analysis, and the expression was normalized to that of *GAPDH*. The results represent the mean ± S.D. of three independent experiments performed in triplicate. *, P<0.05. D. HeLa cells were treated with si-YTHDF2 or si-Control for 48 h. Cells were lysed and subjected to western blot analyses using antibodies against YTHDF2, NICD, HES1, HES5, and β-actin. The membranes were analyzed using ImageJ software (NIH, Bethesda, NY, USA).

To address the question of whether YTHDF2 can act as a Notch signaling regulator, we conducted a qRT-PCR analysis in HeLa cells transiently transfected with vectors encoding the YTHDF2. This detects the transcription of *Hes1*, *Hes5*, and *Notch1*. These genes harbor CSL-binding DNA sequences on their promoters and are essential targets of Notch signaling in epithelial cells 19. Transiently expressed YTHDF2 had a significant inhibitory effect on the mRNA expression of *Notch1*, *Hes1*, and *Hes5* (Figure 1A), indicating that Notch signaling can be downregulated. We confirmed that ectopically expressed-YTHDF2 affects Notch1 and target gene expression through the western blot experiment (Figure 1B). To verify that endogenously expressed YTHDF2 regulates Notch signaling, we performed loss-of-function experiments using siRNA against YTHDF2 (si-YTHDF2). Treatment of HeLa cells with si-YTHDF2 effectively downregulated the expression of YTHDF2 (Figure 1C and 1D). Reduced YTHDF2 significantly upregulated the expression of the Notch1 target genes *Notch1*, *Hes1*, and *Hes5* in transcription (Figure 1C) and resulted in increased translation levels (Figure 1D). Not surprisingly, the amount of NICD, a Notch effector, has also increased. These results suggest that YTHDF2 directly regulates Notch signaling.

### Inhibition of Notch1 signal by YTHDF2 is correlated with the m^6^A value

m^6^A, as one of the most abundant modifications of eukaryotic mRNA, can control any aspect of mRNA post-transcriptional regulation 7, 25. In mammals, m^6^A formation is catalyzed by a nuclear multicomponent complex of two methyltransferases METTL3 and METTL14, as m^6^A writers 7, 25. METTL3 in the complex is the only catalytic component, whereas METTL14 functions in conformational stabilization and RNA substrate recognition 7, 25. The fate of m^6^A -RNA is dependent on m^6^A selective binding proteins 4. Upon most studies about YTHDF2, YTHDF2 regulates the target mRNA level in the cytoplasm. This process is that the YTH domain can selectively bind m^6^A within a G(m^6^A)C consensus site 2, 5. Simultaneously, the P/Q/N-rich N terminus of YTHDF2 can bring the target mRNA to cytoplasmic foci and recruit the CCR4-NOT deadenylase complex inducing RNA degradation 6, 26. Also, HRSP12 has been reported as an adopter protein to bridge YTHDF2 and RNase P/MRP complex that promotes RNA degradation 27.

**Figure 2:**
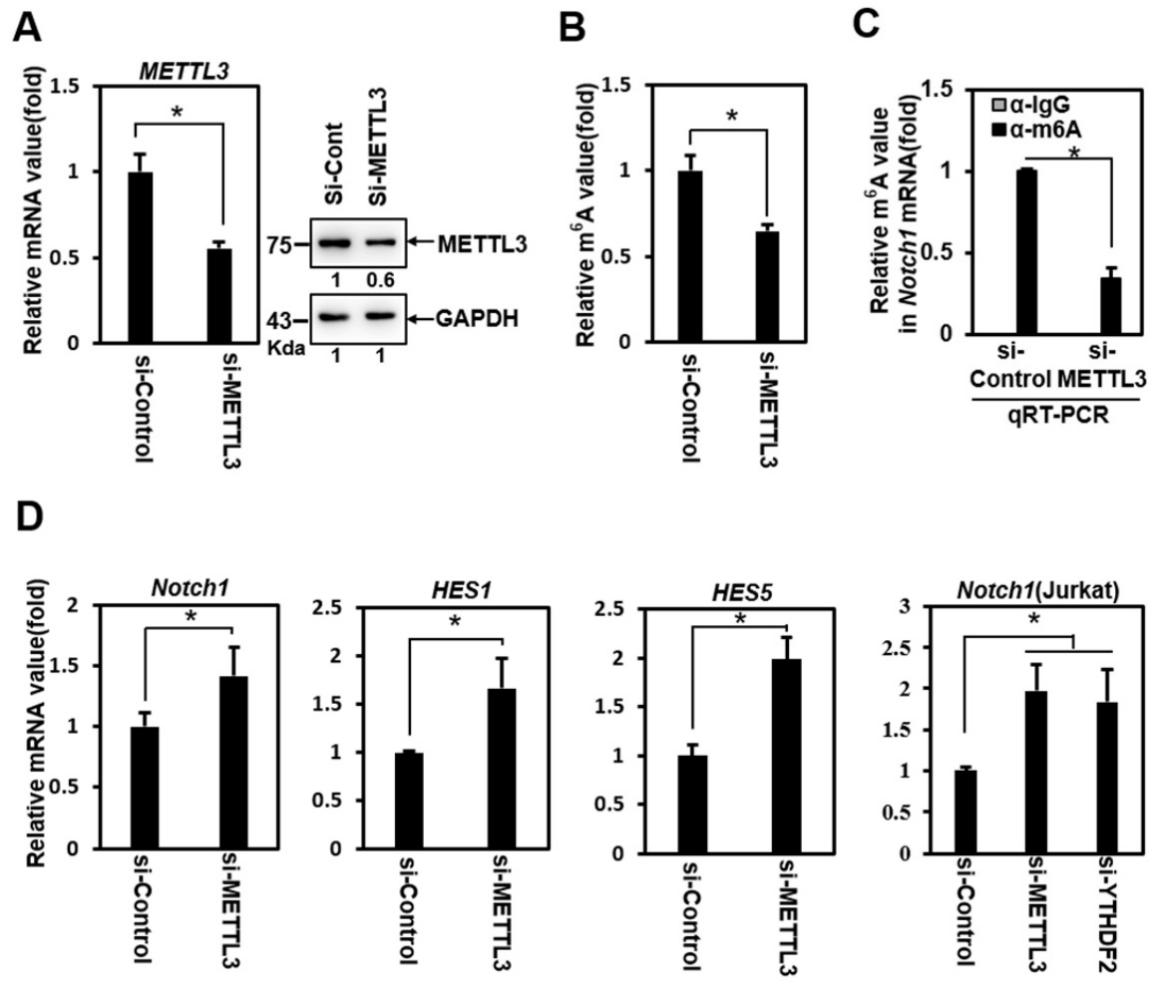
Inhibition of Notch1 signal by YTHDF2 is correlated through the m^6^A value. A. HeLa cells were treated with siRNA (100nM) against *METTL3* (si-METTL3) or control (si-Control) for 48 h. Cells were lysed and subjected to western blot analyses. The membranes were analyzed using ImageJ software (NIH, Bethesda, NY, USA). B. HeLa cells were treated with si-Control (100nM) or si-METTL3 (100nM) for 48 h. Cells were lysed and subjected to m^6^A RNA Methylation Quantification Kit. The results represent the mean ± S.D. of three independent experiments performed in triplicate. *, P<0.05. C. HeLa cells were treated with siRNA (100nM) against *METTL3* (si-METTL3) or control (si-Control) for 48 h. Cells were lysed and subjected to RNA immunoprecipitation using anti- m^6^A Ab. Precipitated RNA was isolated and subjected to qRT-PCR analysis. The results represent the mean ± S.D. of three independent experiments performed in triplicate. *, P<0.05. D. Total RNA was isolated and subjected to qRT-PCR analysis in HeLa cells or Jurkat cells, and the expression was normalized to that of *GAPDH*. The results represent the mean ± S.D. of three independent experiments performed in triplicate. *, P<0.05.

To determine whether YTHDF2 modulates Notch signaling through YTHDF2- m^6^A binding, we performed loss-of-function experiments using siRNA against METTL3 (si-METTL3). Treatment of HeLa cells with si-METTL3 effectively downregulated the expression of METTL3 (Figure 2A) and decreased the m^6^A value of total mRNA (Figure 2B). A relative decrease in the Notch1 m^6^A value was confirmed in addition to the reduced m^6^A value of total mRNA after si-METTL3 treatment in RNA immunoprecipitation experiments using anti- m^6^A Ab (Figure 2C).

Knockdown of METTL3 significantly upregulated the expression of Notch1 target genes *Notch1*, *Hes1*, and *Hes5* (Figure 2D). We also observed this phenomenon in Jurkat cells (Fig. 2D), a T-cell acute lymphoblastic leukemia (T-ALL) cell line driven by aberrant Notch signaling 28. Although this cell has a mutation in Notch itself, resulting in the constitutive active Notch, the treatment of si-YTHDF2 or si-METTL3 effectively upregulated the expression of Notch1 (Figure 2D). These results show the Notch1 target genes upregulation similar to that of the YTHDF2 knockdown experiment, suggesting that YTHDF2 modulates the Notch signal through m^6^A recognition.

### The YTH domain of YTHDF2 is required for the inhibition of Notch signaling

**Figure 3:**
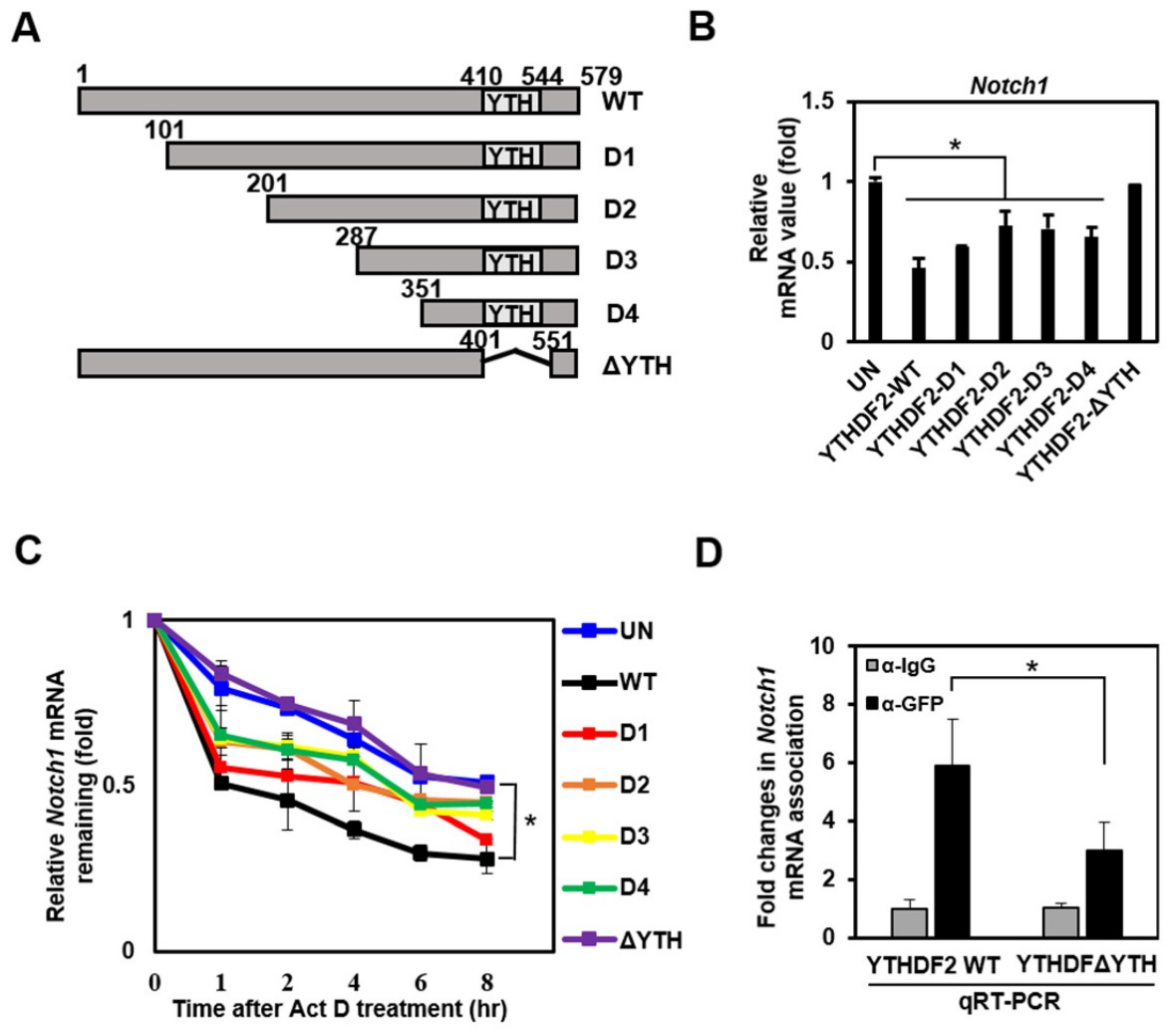
The YTH domain of YTHDF2 is required for the inhibition of Notch signaling. A. Diagram of construction of YTHDF2 deletion mutants. Each YTHDF2 deletion fragment was amplified from YTHDF2 cDNA by PCR and inserted into the restriction enzyme site of pEGFP-C1. B. HeLa cells are transiently transfected with expression vectors encoding YTHDF2 or YTHDF2 deletion mutants. Total RNA was isolated and subjected to qRT-PCR analysis for *Notch1* mRNA. Data were normalized to *GAPDH* expression. The results represent the mean ± S.D. of three independent experiments performed in triplicate. *, P <0.05. C. HeLa cells were transiently transfected with expression vectors encoding YTHDF2 or YTHDF2 deletion mutants, and cells were treated with 10 μg/ml of actinomycin D (Act D) by indicated times. Total RNA was isolated and subjected to qRT-PCR analysis for *Notch1* mRNA. Data were normalized to *GAPDH* expression. The results represent the mean ± S.D. of three independent experiments performed in triplicate. D. HeLa cells were transiently transfected with vectors encoding the YTHDF2-GFP or YTHDF2-ΔYTH-GFP. Cells were lysed and subjected to RNA immunoprecipitation using anti-GFP Ab. Precipitated RNA was isolated and subjected to qRT-PCR analysis. The results represent the mean ± S.D. of three independent experiments performed in triplicate.

To investigate the molecular mechanism underlying the downregulation of Notch target genes by YTHDF2, we constructed several deletion mutants of YTHDF2 followed by N-terminus linking to GFP tags (Figure 3A). We conducted a qRT-PCR analysis that detects the Notch1 transcript to examine these mutants' effect on Notch signaling. Mutants D1, D2, D3, and D4 have a slightly reduced inhibitory activity on Notch1 expression than wild-type YTHDF2. In contrast, the ΔYTH mutation does not reduce *Notch1* mRNA levels (Figure 3B). This suggests that the YTH domain, known to bind m^6^A, has a significant regulatory effect on *Notch1* mRNA levels.

To investigate whether the change in *Notch1* mRNA levels caused by YTHDF2 is due to mRNA degradation, we analyzed mRNA level after stopping new mRNA synthesis by Actinomycin D. The wild type shows the effect on mRNA decay over time, and the other mutants (D1, D2, D3, and D4) show slightly less degradation of mRNA. Still, the ΔYTH mutation does not affect *Notch1* mRNA degradation as in control (UN) (Figure 3C). These results are expected through direct binding of the YTH domain of YTHDF2 to m^6^A on *Notch1* mRNA. Experiments to confirm YTH domain binding to *Notch1*-m^6^A using RNA immunoprecipitation show that the ΔYTH mutant cannot bind *Notch1* mRNA in contrast to WT (Figure 3D).

### The nuclear translocation of YTHDF2 upon Heat shock stress restores Notch signal

**Figure 4:**
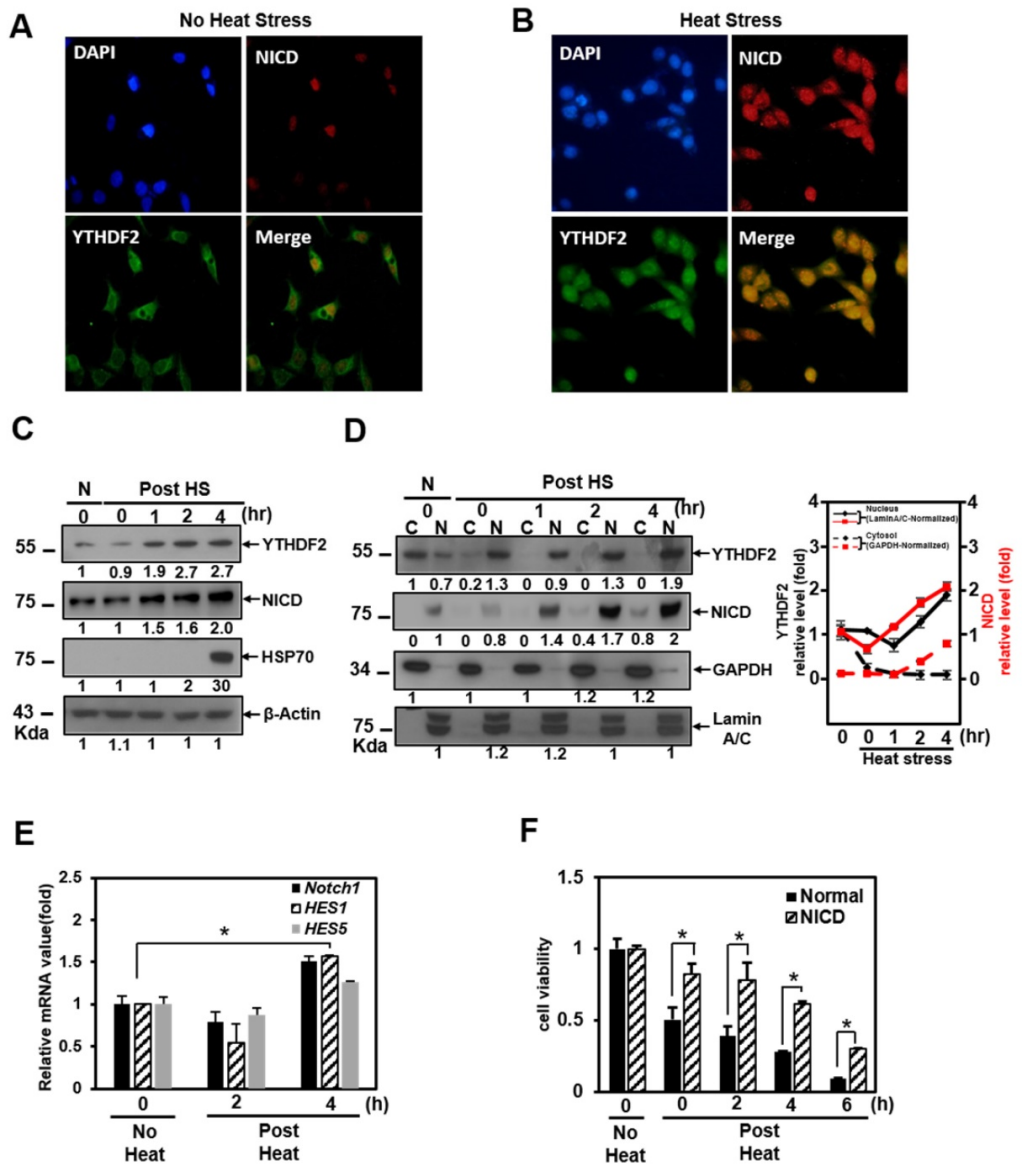

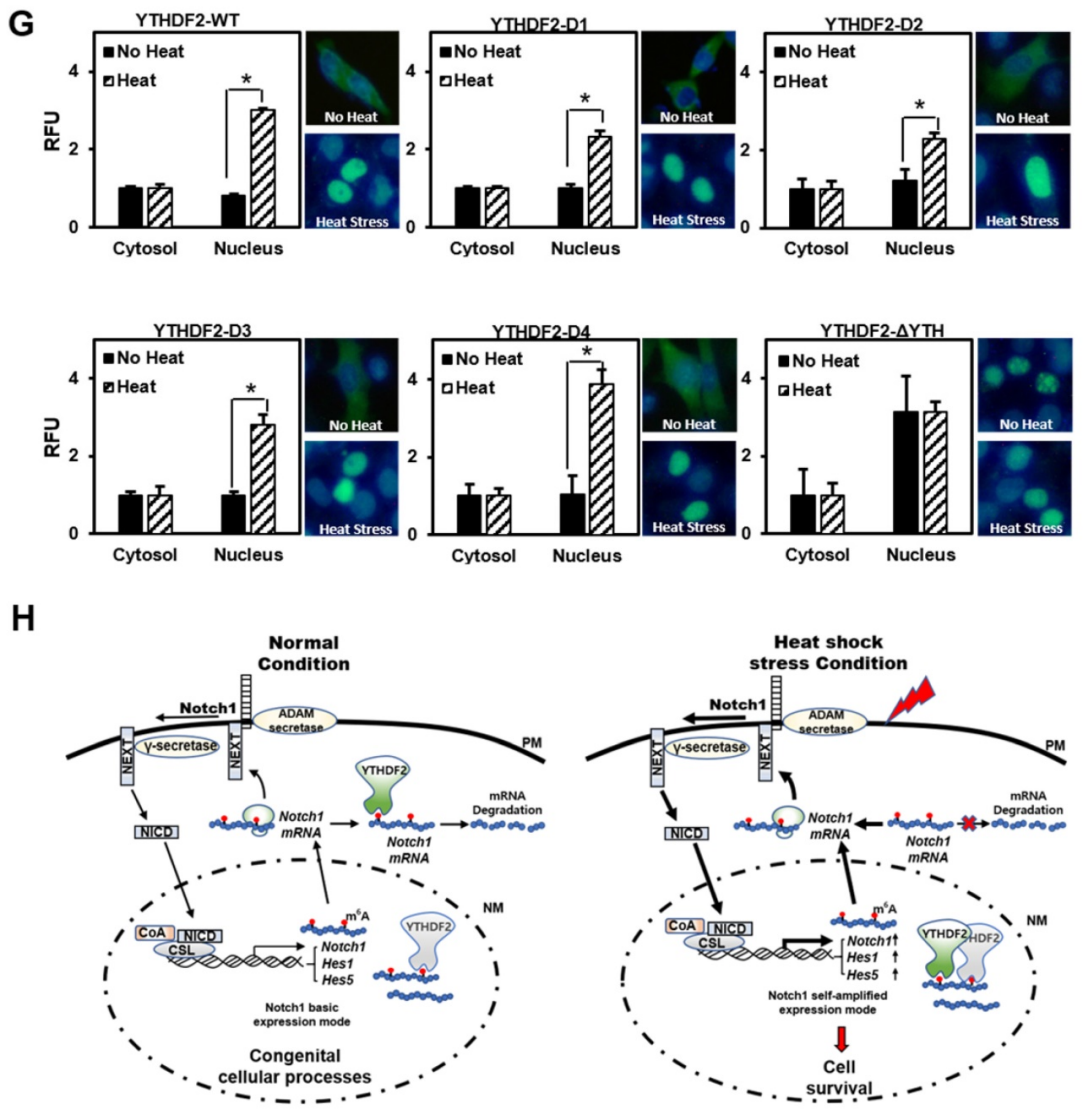
Effect of YTHDF2 Nuclear Translocation by Heat Shock Stress on Notch Signal and Cell Survival. A & B. Subcellular localization of YTHDF2 in HeLa cells before and after heat shock stress. HeLa cells stood 2h After heat shock (42°C, 1h) and were fixed and incubated with NICD antibody and YTHDF2 antibody. Cells were washed and incubated with FITC (for YTHDF2 Ab) or TRITC-conjugated secondary antibodies (for NICD Ab). After staining with DAPI, each fluorescence image merged to show the location of proteins in the cytoplasm and the nucleus. C. Immunoblotting of HeLa cells at the indicated time after heat shock stress (42°C, 1h). N, no heat shock stress. The intensity of the protein bands was analyzed using ImageJ software (NIH, Bethesda, NY, USA). D. HeLa cells were treated after heat shock stress (42°C, 1h). Cells were fractionated and subjected to western blot analyses. Fractionation was verified by using nuclear lamin A/C antibody (nuclear fraction) or GAPDH antibody (cytosolic fraction). The intensity of each fractional protein band was analyzed using ImageJ software (NIH, Bethesda, NY, USA). Relative amounts of nuclear and cytoplasmic NICD and YTHDF2 proteins in four replicates of fractionation experiments were graphed and normalized to nuclear lamin A/C and GAPDH, respectively. E. HeLa cells in Fig. 4C (42°C, 1h) were used for RNA extraction and qRT-PCR. Relative levels of indicated transcripts are normalized to *GAPDH*. The results represent the means ± S.D. of three independent experiments performed in triplicate. *, P<0.05. F. HeLa cells were transfected with NICD for 48 h and then treated with heat shock stress (42°C, 1h). Cells were assayed with MTT at the indicated time. The results represent the means ± S.D. of three independent experiments performed in triplicate. *, P<0.05. G. Relative values of Subcellular localization of YTHDF2 or YTHDF2 mutants in HeLa cells after heat shock stress (42 °C, 1 h). HeLa cells were transfected with 0.5 μg of GFP fused YTHDF2 WT or Mutants expression vectors. Standing for 2 h after heat shock (42 °C, 1 h), cells were fixed and stained with DAPI. Measurements were expressed relative to the number of fluorescent cells. Among cells with fluorescence in a unit area, the value when fluorescence exists only in the cytoplasm was set to the basic value, and the value when it exists in the nucleus was calculated. The results represent the means ± S.D. of three independent experiments performed in triplicate. *, P<0.05. H. A proposed model for YTHDF2 regulating Notch signal in response to heat shock stress. The illustration shows that YTHDF2-mediated *Notch1* mRNA decay suppresses the expression of Notch target genes through YTH domain binding of m^6^A RNA. Under heat stress, YTHDF2 migrates to the nucleus and can restore the expression of Notch target genes required for cell survival and proliferation. CSL, CBF1/suppressor hairless/Lag-1; NEXT, Notch1 extracellular truncation; ADAM, a disintegrin and metalloproteinase; CoA, coactivators; CoR, corepressors; PM, plasma membrane; NM, nuclear membrane

It is well known that Notch signaling is regulated both transcriptionally and post-translationally through the regulation of functional mediators 29. Recently, it has been reported that YTHDF2 migrates to the nucleus faster upon heat shock stress compared to evenly distributed YTHDF2 in the cytoplasm and nucleus under normal cellular conditions 22. But the physiological function of this phenomenon has not been reported. Based on these facts, we performed immunocytochemical staining and cell fractionation with HeLa cells to explore changes in Notch signaling due to nuclear translocation of YTHDF2 under heat shock stress conditions. In our experiments, most YTHDF2 migrates from the cytoplasm to the nucleus in response to heat shock stress (Figure 4A and 4B). Western blots of cell fraction also show that the majority of YTHDF2 is present in the nucleus after heat shock and maintains similar amounts for up to 4 h or more in Figure 4D.

We found that YTHDF2 increased in a pattern similar to that of Hsp70 and that NICD started to increase 1 hour after heat shock and clearly increased after 2 h in Western blots showing the expression levels of each protein (Fig. 4C). On the other hand, after heat shock, we found no significant difference in the proportion of NICD in the cytoplasm and nucleus (Figure 4D). It is known that NICD, a Notch effector, is post-translationally regulated by ubiquitination for the normal proteasome proteolytic process 30. We tested the probability that the increase in the amount of NICD protein after heat shock is due to the decreased ubiquitination of NICD. However, there was no significant difference in the amount of ubiquitin-tagged NICD in the cytoplasm before and after heat shock (data not shown). The increase in NICD appears to be due to a relative decrease in cytoplasmic YTHDF2 after heat shock. This phenomenon is consistent with YTHDF2 depletion increasing the mRNA expression level of Notch1, as shown in the YTHDF2 knockdown experiment in Figure 1C.

As shown in the cell fractionation experiments, YTHDF2 is present in the nucleus after heat shock. In contrast, there is no significant difference in the proportion of NICD in the cytoplasm and nucleus after heat shock (Figure 4D). This phenomenon means that nuclear translocation of YTHDF2 delays the Notch1 mRNA decay under heat shock conditions, indicating that NICD protein, acting as a transcriptional activator on target genes in the nucleus, also increases proportion to the increase in total intracellular NICD (Figure 4C and 4D). To reinforce this explanation, we confirmed that the NICD target genes *Notch1*, *hes1*, and *hes5* are increased under the same heat shock stress conditions (Figure 4E). Furthermore, the viability of exogenous NICD-expressing cells is significantly higher than that of the control group up to 6 h after heat shock stress (Figure 4F). These experimental results suggest that YTHDF2 located in the nucleus is less involved in the breakdown of *Notch1* mRNA in the cytoplasm after heat shock. The increased NICD in the cytoplasm finally induces the target gene expression, thereby increasing the cell viability. We express these proposals using a simple schematic model in Figure 4H.

### The YTH domain of YTHDF2 is required for nuclear translocation of YTHDF2 upon Heat shock stress

To determine which part is involved in the migration of YTHDF2 to the nucleus by heat shock stress, we investigated the intracellular distribution of the GFP-YTHDF2 deleted mutants, as shown in Figure 3A. Our results show that mutants D1, D2, D3, and D4 migrate to the nucleus after heat shock without much difference than wild-type YTHDF2 (Figure 4G). Specifically, we found that mutant ΔYTH is already more distributed in the nucleus before heat shock than in other mutants. No change in translocation is observed even after heat shock (Figure 4G). These results show that the YTH domain involving the RNA stability regulation is necessary for YTHDF2 to exist in the cytoplasm in normal conditions. It is also required for moving to the nucleus under heat shock stress conditions. Our results imply that other YTHDF2-binding proteins that participate in the migration to the nucleus might be in the cytoplasm. In particular, further studies on the cargo protein that binds around the YTH domain are needed. In this study, we suggest for the first time that the re-localization of YTHDF2 due to heat shock stress may be affected by the presence or absence of the YTH domain.

## Conclusion

The study found that YTHDF2 suppresses the Notch1 expression by modulating *Notch1* mRNA stability through the YTH domain binding of Notch1 m^6^A RNA. Our results show that YTHDF2 migrates to the nucleus, restoring the Notch1 expression required for cell survival and proliferation in response to extracellular stress as heat shock. This study is vitally important as it adds a new concept, post-transcriptional regulation, to the basic transcriptional and post-translational regulation of Notch signaling. All these results are summarized in the legend in Figure 4H. Our findings suggest that YTHDF2 can post-transcriptionally regulate *Notch1* mRNA, which acts as an intrinsic repressor of Notch signaling.

## Supplementary Material

Supplementary tables.

## Abbreviations

YTHDF2: YTH domain Family2
YTH: YT521-B homology
m^6^A: N^6^-methyladenosine
CSL: CBF1/suppressor hairless/Lag-1
NEXT: Notch1 extracellular truncation
NICD: Notch intracellular domain
ADAM: A disintegrin and metalloproteinase
CoA: Coactivators
CoR: Corepressors
