# Supplementary material for: YTHDF2 Suppresses Notch Signaling through Post-transcriptional Regulation on Notch1
Source: Int J Biol Sci. 2021 Aug 28;17(14):3776–85. doi: 10.7150/ijbs.61573 (PMC8495403; doi:10.7150/ijbs.61573)
Supplement: Supplementary file 1 — Supplementary tables. [file ijbsv17p3776s1.pdf]

Supplementary Table. 1 Alignment of Notch1 homologs between Human and Zebrafish by Clustal Omega

```
CLUSTAL O(1.2.4) multiple sequence alignment


Human      MPPLLAPLLCL-ALLPALAARGPRCSQPGETCLNGGKCEAA-NGTEACVCGGAFVGPRCQ 58
Zebra      MNRFLVKLTLLTAASLATVAQGQRC---SEYCQNGGICEYKPSGEASCRCPADFVGAQCQ 57
           *  :*. *  * *   * .*:* **   .* * *** **   .*  :* * . *** :**

Human      DPNPCLSTPCKNAGTCHVVDRRGVADYACSCALGFSGPLCLTPLDNACLTNPCRNGGTCD 118
Zebra      FPNPCNPSPCRNGGVCRPQMQGNEVGVKCDCVLGFSDRLCLTPVNHACMNSPCRNGGTCS 117
            ****  :**:*.*.*:    :  . ..   *.*.****. *****:::**:..********.

Human      LLTLTEYKCRCPPGWSGKSCQQADPCASNPCANGGQCLPFEASYICHCPPSFHGPTCRQD 178
Zebra      LLTLDTFTCRCQPGWSGKTCQLADPCASNPCANGGQCSAFESHYICTCPPNFHGQTCRQD 177
           ****  :.*** ******:** ***************  **: *** ***.*** *****

Human      VNECGQKPGLCRHGGTCHNEVGSYRCVCRATHTGPNCERPYVPCSPSPCQNGGTCRPTGD 238
Zebra      VNECAVSPSPCRNGGTCINEVGSYLCRCPPEYTGPHCQRLYQPCLPSPCRSGGTCVQTSD 237
           ****.  .*.  **:**** ****** * *    :***:*:* * ** ****:.****  *.*

Human      VTHECACLPGFTGQNCEENIDDCPGNNCKNGGACVDGVNTYNCRCPPEWTGQYCTEDVDE 298
Zebra      TTHTCSCLPGFTGQTCEHNVDDCTQHACENGGPCIDGINTYNCHCDKHWTGQYCTEDVDE 297
           .** *:********.**.*:***   : *:*** *:**:*****:*  .************

Human      CQLMPNACQNGGTCHNTHGGYNCVCVNGWTGEDCSENIDDCASAACFHGATCHDRVASFY 358
Zebra      CELSPNACQNGGTCHNTIGGFHCVCVNGWTGDDCSENIDDCASAACSHGATCHDRVASFF 357
           *:* ************* **::**********:*************** ************:

Human      CECPHGRTGLLCHLNDACISNPCNEGSNCDTNPVNGKAICTCPSGYTGPACSQDVDECSL 418
Zebra      CECPHGRTGLLCHLDDACISNPCQKGSNCDTNPVSGKAICTCPPGYTGSACNQDIDECSL 417
           **************:*********::**********.******** **** **.**:******

Human      GANPCEHAGKCINTLGSFECQCLQGYTGPRCEIDVNECVSNPCQNDATCLDQIGEFQCIC 478
Zebra      GANPCEHGGRCLNTKGSFQCKCLQGYEGPRCEMDVNECKSNPCQNDATCLDQIGGFHCIC 477
           *******.*:*:** ***:*:***** *****:****** ***************** *:***

Human      MPGYEGVHCEVNTDECASSPCLHNGRCLDKINEFQCECPTGFTGHLCQYDVDECASTPCK 538
Zebra      MPGYEGVFCQINSDDCASQPCL-NGKCIDKINSFHCECPKGFSGSLCQVDVDECASTPCK 536
           *******.*::*:*:***.*** **:*:****.*:*****.**:*.**:* *** ***********

Human      NGAKCLDGPNTYTCVCTEGYTGTHCEVDIDECDPDPCHYGSCKDGVATFTCLCRPGYTGH 598
Zebra      NGAKCTDGPNKYTCECTPGFSGIHCELDINECASSPCHYGVCRDGVASFTCDCRPGYTGR 596
           ***** ****.*** ** *::* ***:**:**   .***** *:*****:*** *******:

Human      HCETNINECSSQPCRHGGTCQDRDNAYLCFCLKGTTGPNCEINLDDCASSPCDSGTCLDK 658
Zebra      LCETNINECLSQPCRNGGTCQDRENAYICTCPKGTTGVNCEINIDDCKRKPCDYGKCIDK 656
            ******** *****:********:***:* * ***** *****:***   .*** *.*:**

Human      IDGYECACEPGYTGSMCNINIDECAGNPCHNGGTCEDGINGFTCRCPEGYHDPTCLSEVN 718
Zebra      INGYECVCEPGYSGSMCNINIDDCALNPCHNGGTCIDGVNSFTCLCPDGFRDATCLSQHN 716
           *:****.*****:*********:** ********* **:*.*** **:*::*.****: *

Human      ECNSNPCVHGACRDSLNGYKCDCDPGWSGTNCDINNNECESNPCVNGGTCKDMTSGYVCT 778
Zebra      ECSSNPCIHGSCLDQINSYRCVCEAGWMGRNCDININECLSNPCVNGGTCKDMTSGYLCT 776
           **.****:**:* *.:*.*:* *: ** * ***** *** *****************:**

Human      CREGFSGPNCQTNINECASNPCLNQGTCIDDVAGYKCNCLLPYTGATCEVVLAPCAPSPC 838
Zebra      CRAGFSGPNCQMNINECASNPCLNQGSCIDDVAGFKCNCMLPYTGEVCENVLAPCSPRPC 836
           ** ******** **************:*******:****:***** .** *****:* **

Human      TQYRCECPSGWTGLYCDVPSVSCEVAAQRQGVDVARLCQHGGLCVDAGNTHHCRCQAGYT 1138
Zebra      ASFTCQCASGWTGIYCDVPSVSCEVAARQQGVSVAVLCRHAGQCVDAGNTHLCRCQAGYT 1136
           :.: *:* *****:**************::***.** **:*.* ********* ********

Human      GSYCEDLVDECSPSPCQNGATCTDYLGGYSCKCVAGYHGVNCSEEIDECLSHPCQNGGTC 1198
Zebra      GSYCQEQVDECQPNPCQNGATCTDYLGGYSCECVPGYHGMNCSKEINECLSQPCQNGGTC 1196
           ****:: ****.*.*****************:** ****:***:**:****:********

Human      LDLPNTYKCSCPRGTQGVHCEINVDDCNPPVDPVSRSPKCFNNGTCVDQVGGYSCTCPPG 1258
Zebra      IDLVNTYKCSCPRGTQGVHCEIDIDDCSPSVDPLTGEPRCFNGGRCVDRVGGYGCVCPAG 1256
           :** ******************::***.*.***: .*:***.* ***:****.*.**.* *

Human      FVGERCEGDVNECLSNPCDARGTQNCVQRVNDFHCECRAGHTGRRCESVINGCKGKPCKN 1318
Zebra      FVGERCEGDVNECLSDPCDPSGSYNCVQLINDFRCECRTGYTGKRCETVFNGCKDTPCKN 1316
           ****************:***  *: **** :***:****:*:**:***:*:****..****

Human      GGTCAVASNTARGFICKCPAGFEGATCENDARTCGSLRCLNGGTCISGPRSPTCLCLGPF 1378
Zebra      GGTCAVASNTKHGYICKCQPGYSGSSCEYDSQSCGSLRCRNGATCVSGHLSPRCLCAPGF 1376
           ********** :*:****  *:.*::** *:::****** **.**:**  ** ***    *

Human      TGPECQFPASSPCLGGNPCYNQGTCEPTSESPFYRCLCPAKFNGLLCHILDYSFGGGAGR 1438
Zebra      SGHECQTRMDSPCLV-NPCYNGGTCQPISDAPFYRCSCPANFNGLLCHILDYSFSGGQGR 1435
           :* ***   .****  ***** ***:* *:.:***** ***:*************.** **

Human      DIPPPL-IEEACELPECQEDAGNKVCSLQCNNHACGWDGGDCSLNFNDPWKNCTQSLQCW 1497
Zebra      DIAPPVEVEIRCEIAQCEGRGGNAICDTQCNNHACGWDGGDCSLNFDDPWQNCSAALQCW 1495
           ** **: :*  **: :*:  .** :*. ******************:***:**::  :****

Human      KYFSDGHCDSQCNSAGCLFDGFDCQRAEGQCNPLYDQYCKDHFSDGHCDQGCNSAECEWD 1557
Zebra      RYFNDGKCDEQCATAGCLYDGFDCQRLEGQCNPLYDQYCRDHYADGHCDQGCNNAECEWD 1555
           :**.**:**.** :****:******* ************:**::*********.******

Human      GLDCAEHVPERLAAGTLVVVVLMPPEQLRNSSFHFLRELSRVLHTNVVFKRDAHGQQMIF 1617
Zebra      GLDCADDVPQKLAVGSLVLVVHIPPDELRNRSSSFLRELSSLLHTNVVFRRDANGEALIF 1615
           *****:.**::**.*:**:** :**::***  *  ****** :*******:***:*: :**

Human      PYYGREEELRKHPIKRAAEGWAAPDALLGQVKASLLPGGSEGGRRRRELDPMDVRGSIVY 1677
Zebra      PYYGSEHELSKHK----RSDWTDPGQLMQRARRSLTS--FLKPRTRRELDHMEVKGSIVY 1669
           **** *.** **      ..*: *. *: :.: **         * ***** *:*:*****

Human      LEIDNRQCVQASSQCFQSATDVAAFLGALASLGSLNIPYKIEAVQSETVEPPPPAQLHFM 1737
Zebra      LEIDNRQCFQQSDECFQSATDVAAFLGALASSGNLNVPYIIEAVTSEGG-PPKTGEMYPM 1728
           ********.* *.:****************** *.**:** **** **    **   .::: *

Human      YVAAAAFVLLFFVGCGVLLSRKRRRQHGQLWFPEGFKVSEASKKKRREPLGEDSVGLKPL 1797
Zebra      FLVLLALAVLALAAVGVVVSRKRKREHGQLWFPEGFKVNEP-KKKRREPVGEDSVGLKPL 1787
           ::.  *:.:* :.. **::****:*:************.*  *******:**********

Human      KNASDGALMDDNQNEWGDEDLETKKFRFEEPVVLPDLDDQTDHRQWTQQHLDAADLRMSA 1857
Zebra      KNS-DSSLMDEQLSEWAEDDT-NKRFRFEGQSIL-EMSGQLDHRQWTQQHLDAADLRLNS 1844
           **: *.:***:: .**.::*  .*:****   :* ::..* ****************:.:

Human      MAPTPPQGEVDADCMDVNVRGPDGFTPLMIASCSGGGLETGNSEEEED-APAVISDFIYQ 1916
Zebra      MAPTPPQGQIENDCMDVNVRGPDGFTPLMIASCSGGGLENENGEAEEDPSADVITDFIYH 1904
           ********::: *************************. *.* *** :  **:****:

Human      GASLHNQTDRTGETALHLAARYSRSDAAKRLLEASADANIQDNMGRTPLHAAVSADAQGV 1976
Zebra      GANLHNQTDRTGETALHLAARYARSDAAKRLLESCADANVQDNMGRTPLHAAVAADAQGV 1964
           **.*******************:**********:.****:*************:******

Human      FQILIRNRATDLDARMHDGTTPLILAARLAVEGMLEDLINSHADVNAVDDLGKSALHWAA 2036
Zebra      FQILIRNRATDLDARMHDGTTPLILATRLAVEGMVEELINCHADPNAVDDSGKSALHWAA 2024
           **************************:*******:*:***.*** ***** *********

Human      AVNNVDAAVVLLKNGANKDMQNNREETPLFLAAREGSYETAKVLLDHFANRDITDHMDRL 2096
Zebra      AVNNVDAAVVLLKNGANKDLQNNKEETPLFLAAREGSYETAKVLLDHLANRDIADHLDQL 2084
           *******************:***:***************************:*****:**:*:*

Human      PRDIAQERMHHDIVRLLDEYNLVRSPQLHGAPLGGTPTLSPPLCSPNGYLGSLKPG---- 2152
Zebra      PRDIAHERMHHDIVRLLEEYNLVRSPPL---------PLSPPLCCPNTYLGIKPSPGNNN 2135
           *****:***********:******** *         ******.** ***

Human      VQGKKVRKPSSKGLACGSKEAKDLKARRKKSQDGKGCLLDSSGMLSPVDSLESPHGYLSD 2212
Zebra      NTAKKTRKPGGKGVG-GKDSGKDIRTKKKKSGDGKNGGIMEVGVLSPVDSLESPHGYLSD 2194
             .**.***..**:. *....**::::*** ***.  : . *:****************

Human      VASPPLLPSPFQQSPSVPLNHLPGMPDTHLGIGHLNVAAKPEMAALGGGGRLAFETGPPR 2272
Zebra      VSSPPMMTSPFQQSPPISLNQLQGLADSHMGGALQGLG-------------KPF-DSAPR 2240
           *:***:: ******* : **:* *: *:*:* .  .:.             *  . **

Human      LSHLPVASGTSTVLGSSSGGALNFTVGGSTSLNGQCEWLSRLQSGMVPNQYNPLRGSVAP 2332
Zebra      LSHLPVANNVG------------------GAQAGACDWLQRVQQQQQQQQQQQAGMLMP 2282
           *******....                  :  * *:**.*:*.    :* :   * : *

Human      GPLSTQAPSLQHGMVGPLHSSLAASALSQMMSYQGLPSTRLATQPHLVQTQQVQPQNLQM 2392
Zebra      ------------------TMLSATNMPQVMGYPTMQSSHLGAPSHMIAHQNMAPMQH-- 2321
                             : *:*: : *:*.* : *::*.:  *::  *:: * :

Human      QQQNLQPANIQQQQSLQPPPPPPQPHLGVSSAASGHLGRSFLSGEPSQADVQPLGPSSLA 2452
Zebra      --QNIS----------------------H--------HFL-GDLSGLDLQS-SSGHAP 2345
             **:.                              ** *: *  *:*  . .

Human      VHTILPQESPALPTSLPSSLVPPVTAAQFLTPPSQHSYSSPVDNTPSHQLQVPEHPFLTP 2512
Zebra      IQTILPQDS--------QRMAPPISSTQFLTPPSQHSYSNPMDNTPNHQ-QVPDHPFLTP 2398
           ::*****:*        . :.**::::***********.*:****.** ***:******

Human      SPESPDQWSSSSPHSNVSDWSEGVSSPPTSMQSQIARIPEAFK  2555
Zebra      SAGSPDQWSSSSPHSNLSDWSEGISSPPTSMQ--MNHIPEAFK  2437
           *  *************:******:********  : :******
```

Supplementary Table. 2 Alignment of YTHDF2 homologs between Human and Zebrafish by Clustal Omega

```
CLUSTAL O(1.2.4) multiple sequence alignment


Human           MSASSLLEQRPKGQGNKVQNGSVHQKDGLNDDDFEPYLSPQARPNNAYTAMSDSYLPSYY     60
Zebrafish       MSASSLLEQRPKGQANKVQNGAVTQKDTLNDDEFEPYLNAQPRQSNAYTAMSDSYMPSYY     60
                **************.******:* *** ****:*****. * * .**********:****

Human           SPSIGFSYSLGEAAWSTGGDTAMPYLTSYGQLSNGEPHFLPDAMFGQPGALGSTPFLGQH     120
Zebrafish       SPSIGFTYSLNEAAWSTGGDPPMPYLASYGQLSNGEHHFLPDAMFGQSGALGNNPFLGQH     120
                ******:***.*********  ****:********* ********** ****..******

Human           GFNFFPSGIDFSAWGNNSSQGQSTQSSGYSSNYAYAPSSLGGAMIDGQSAFA-NETLNKA     179
Zebrafish       GFNFFPSGIDFPAWGNSSSQGQSTQSSGYSSSYAYAPSTLGGAMIDGQSPFAANEPLNKA     180
                *********** ****.**************.******:********** ** ** ****

Human           PGMNTIDQGMAALKLGSTEVASNVPKVVGSAVGSGSITSNIVASNSLPPATIAPPKPASW     239
Zebrafish       VGMNSLDQGMAGLKIGAGDMA---PKVVGSGLPGGPL-SQVSAAPTMPPASMAPAKTASW     238
                 ***::*****.**:*: ::*   ******.: .* : *:: *: ::***::** * ***

Human           ADIASKPAKQQPKLKTKNGIAGSSLPPPPIKHNMDIGTWDNKGPVAKAP--SQALVQNIG     297
Zebrafish       ADIASKPAKPQPKLKTKGGLGGTNLPPPPIKHNMDIGTWDNKGNMPKPAAPQQTSLPTNG     298
                ********* *******.*:.*:.******************* : *    .*: : . *

Human           QPT-QGSPQPVGQQANNSPPVAQ------ASVGQQ-TQPLPP------PPPQPAQLSVQQ     343
Zebrafish       QPPNQSSPQPG-ATAGGVPQLPLSNGQLVPPTGQLVQHPLPPGGQPGAVPPQLSQGPPVS     355
                **  *.****    *.. * :         .**   :****       *** :*    .

Human           QAAQPTRWVAPRNRGSGFGHNGV-DGNGVGQSQAGSGSTPSEPHPVLEKLRSINNYNPKD     402
Zebrafish       QPSQPTRWVPPRNRANGFGDAAGGPGQSPPNSGMGGITVPAEPHPVLEKLRMVNNYNPKD     415
                * :****** ****..***. .    *:.  :*  *. :.*:********** :*******

Human           FDWNLKHGRVFIIKSYSEDDIHRSIKYNIWCSTEHGNKRLDAAYRSMNGKGPVYLLFSVN     462
Zebrafish       FDWNPKHGRVFIIKSYSEDDIHRSIKYNIWCSTEHGNKRLDAAYRSLANKGPPYLLFSVN     475
                **** *****************************************: .*** *******

Human           GSGHFCGVAEMKSAVDYNTCAGVWSQDKWKGRFDVRWIFVKDVPNSQLRHIRLENNENKP     522
Zebrafish       GSGHFCGVAEMRSPVDYNTCAGVWSQDKWKGRFDVRWIFVKDVPNSQLRHIRLENNENKP     535
                ***********:* **********************************************

Human           VTNSRDTQEVPLEKAKQVLKIIASYKHTTSIFDDFSHYEKRQEEEESVKKERQGRGK---     579
Zebrafish       VTNSRDTQEVPLDKARQVLKIIASYKHTTSIFDDFSHYEKRQEEEESVKKVTCHGLAPSG     595
                ************:**:**********************************

Human           -          579
Zebrafish       I          596
```
